# Analysis of the correlation between high iodized salt intake and the risk of thyroid nodules: a large retrospective study

**DOI:** 10.1186/s12885-021-08700-z

**Published:** 2021-09-07

**Authors:** Yaohui Wang, Jiangang Wang, Zhihen Chen, Min Ma, Changwei Lin, Qingnan He, Mingzhu Ye

**Affiliations:** 1 Health Management Center, the Third Xiangya Hospital of Central South University, Changsha, 410013 China; 2 Department of Gastrointestinal Surgery, the Third Xiangya Hospital of Central South University, Changsha, 410013 China

**Keywords:** Thyroid nodules, Thyroid cancer, Iodized salt, Risk factor

## Abstract

**Background:**

Currently, whether daily excess iodized salt intake increases the risk of thyroid nodules and even thyroid cancer remains controversial. Our research group aimed to provide a theoretical basis for the clinical guidance of daily iodized salt intake and the prevention of thyroid nodules through a retrospective analysis of the correlation between daily iodized salt intake and the risk of thyroid nodules and thyroid cancer in Hunan, China.

**Methods:**

This study retrospectively analyzed the data of subjects who underwent a physical examination at the Health Management Center, Third Xiangya Hospital of Central South University, between January 1, 2017, and December 31, 2019. Subjects enrolled in this study underwent thyroid ultrasonography and tests to urine routines and liver and kidney function, and all subjects completed a questionnaire survey. The daily iodized salt intake of the study subjects was estimated based on spot urine methods (Tanaka). A multivariate logistic regression model was used to analyze the relationship between daily iodized salt intake and thyroid nodules and thyroid cancer.

**Results:**

Among the 51,637 subjects included in this study, the prevalence of thyroid nodules was 40.25%, and the prevalence of thyroid cancer was 0.76%; among all enrolled subjects, only 3.59% had a daily iodized salt intake less than 5 g. In addition, we found that a daily intake of more than 5 g of iodized salt was not only an independent risk factor for the occurrence of thyroid nodules (odds ratio (OR): 2.08, 95% confidence interval (CI): 1.86–2.31, *p* < 0.001) but also an independent risk factor for the occurrence of thyroid cancer (OR: 5.81, 95% CI: 1.44–23.42, *p* = 0.012). A pooled analysis showed a significantly higher risk of thyroid nodules in subjects aged > 60 years with a daily iodized salt intake of more than 5 g compared to subjects aged < 60 years with a daily iodized salt intake of no more than 5 g (OR: 4.88, 95% CI: 4.29–5.54, *p* < 0.001); the risk of thyroid cancer was not significantly different between subjects aged > 60 years with a daily iodized salt intake of more than 5 g and those aged < 60 years with a daily iodized salt intake of no more than 5 g (OR: 2.15, 95% CI: 0.52–8.95, *p* = 0.281). The risk of thyroid nodules was not increased in physically active subjects with a daily iodized salt intake of more than 5 g compared to physically inactive subjects with a daily iodized salt intake of no more than 5 g (OR: 1.12, 95% CI: 0.97–1.28, *p* = 0.111). The same protective effect of physical activity was observed for thyroid cancer in subjects whose daily iodized salt intake exceeded 5 g. The risk of thyroid nodules was reduced for subjects with an education level of postgraduate and above, even when the daily iodized salt intake exceeded 5 g, compared to those with high school education and below and a daily iodized salt intake of no more than 5 g (OR: 0.79, 95% CI: 0.66–0.93, *p* = 0.005); however, a protective effect of education level on the occurrence of thyroid cancer was not observed. Independent risk factors affecting daily iodized salt intake greater than 5 g included age, triglycerides, family history of tumors, physical activity, and marital status.

**Conclusions:**

Daily intake of more than 5 g of iodized salt increased the risk of thyroid nodules and thyroid cancer, while increased physical activity and education level reduced the risk of thyroid nodules and thyroid cancer caused by iodized salt intake.

**Supplementary Information:**

The online version contains supplementary material available at 10.1186/s12885-021-08700-z.

## Introduction

Thyroid nodules are frequently detected in healthy individuals undergoing physical examinations [1, 2]. The detection rate of thyroid nodules by simple physical examination is 5–7%, and the detection rate of thyroid nodules by combined ultrasound examination can be as high as 20–76% [3]. Based on pathological characteristics, thyroid nodules can be divided into benign nodules and malignant nodules [4]. The majority of thyroid nodules found during a physical examination are benign, and a few are malignant; a small number of benign nodules may further develop into malignant lesions [5, 6]. Many factors, including sex and ionizing radiation, may affect thyroid nodule formation and malignancy [7]. However, whether excessive daily iodized salt increases the risk of thyroid nodules and thyroid cancer remains controversial [8, 9].

Iodine is an essential trace element involved in the synthesis of thyroid hormone. Iodine deficiency disorders affect various stages of human growth and development and cause different clinical manifestations [10]. In particular, iodine deficiency in pregnant women can affect fetal neurodevelopment, leading to mental retardation in newborns. Since 1995, China has implemented a universal salt iodization program. Ten years later, the rate of goiter in Chinese students aged 7–14 decreased from 20.4% to less than 5%; thyroid-related diseases caused by iodine deficiency, including simple goiter, cretinism, mental disorders and neuronal development disorders, were also greatly reduced [11]. During that same time period, some scholars observed an increasing trend in the incidence of thyroid nodules and thyroid cancer, and they suggested that salt iodization was a risk factor for the occurrence and development of thyroid nodules [12, 13]. However, some scholars offered different viewpoints [14]. Chen et al. found that even with salt iodization, the intake of iodine in Zhoushan, China, is still insufficient, and salt iodization is necessary for inland areas [9]. Given that it is unclear whether the daily intake of excess iodized salt will increase the risk of thyroid nodules and thyroid cancer, more clinical data are needed for follow-up studies.

Hunan is located in the hilly area of central China. The residents consume iodized salt, and most residents have a preferred dietary taste for and a large daily intake of iodized salt. Therefore, this study aimed to investigate the correlation between daily iodized salt intake and thyroid nodules and thyroid cancer by retrospectively analyzing the risk factors for thyroid nodules and thyroid cancer in the Hunan region. The risk factors for daily salt intake were further explored to provide scientific suggestions for clinical guidance on daily iodized salt intake and the prevention and treatment of thyroid nodules and thyroid cancer.

## Materials and methods

### Research design

This was a cross-sectional study based on data obtained during physical examinations. The information and test results for all individuals who underwent a physical examination at the Health Management Center, Third Xiangya Hospital of Central South University, between January 1, 2017, and December 31, 2019, were collected. Potential bias was controlled by including all physical examination data recorded for that time period rather than conducting random sampling.

### Study subjects

This study retrospectively collected data of 53,784 healthy subjects who underwent physical examinations at the Health Management Center, Third Xiangya Hospital of Central South University, between January 1, 2017, and December 31, 2019. A total of 2147 subjects had missing clinical data and, thus, were excluded; therefore, the data for 51,637 subjects were included in the final analyses. The subjects included were long-term residents of the Hunan region. This study received ethics approval from the Third Xiangya Hospital of Central South University, and all patients signed informed consent forms.

### Clinical and laboratory data collection

The clinical and laboratory data of each subject, including general information such as body weight and height; laboratory tests, such as fasting blood glucose, blood lipids, and urine sodium; and special tests, such as thyroid ultrasound, were collected by physicians. Body mass index (BMI) was calculated as body weight (kg) divided by square of height (m) (kg/m^2^). Body mass index (BMI) was classified into four grades [15]: BMI < 18.5 was defined as underweight, BMI 18.5–23.9 was defined as normal weight, BMI 24.0–27.9 was de-fined as overweight, and BMI > 28.0 was defined as obese.

### Questionnaire

In addition to the physical examination and laboratory tests, we also designed a questionnaire based on the *National Physical Examination Questionnaire* [16]. The questionnaire included general information, such as sex, age, family history of tumors, history of exposure to hazardous substances, marital status and education level, as well as lifestyle information, such as smoking history, drinking history, physical activity, work hours and sleep duration. The history of exposure to toxic substances included noise, vibration, geomagnetic radiation, chemical pollution, dust, air pollution, cooking fumes, etc. Smokers were defined as those with continuous or cumulative smoking for more than 6 months [17]; drinkers were defined as those whose alcohol intake exceeded 10 g per day [18]; and physical activity was defined as being physically active, at a moderate intensity or higher, 3 or more times per week, lasting at least 30 min each session [19].

### Daily iodized salt intake assessment

Based on literature and previous studies [16, 20, 21], we calculated 24-h urinary sodium excretion using the Tanaka formula to estimate daily iodized salt intake. The calculation formula was as follows: Estimated 24-h urinary sodium excretion =21.98 × ((Naspot÷Crspot) × Pr_24hCr_)^0.392^. Pr_24hCr_ = 14.89 × weight + 16.14 × height-2.04 × age-2244.45. Naspot = spot urinary sodium (mmol/l); Crspot = spot urinary creatinine (mmol/l); Iodized salt = NaCl = Estimated 24-h urinary sodium excretion× 2.55. The urinary sodium test was examined by an ion selective electrode method.

### Diagnosis of thyroid nodules and thyroid cancer

The diagnosis of thyroid nodules relies on thyroid ultrasound through a direct scan with a linear array or trapezoidal high-frequency probe. The probe frequency was 7 ~ 10 MHz, and the size, number and nature of thyroid nodules was evaluated. The diagnosis of thyroid cancer relies on a pathological diagnosis.

### Data analysis

The clinical data and laboratory results of the subjects are expressed as the mean ± standard deviation. The χ2 test was used to compare categorical variables, and analysis of variance was used to compare continuous variables. Logistic regression analysis was performed to evaluate the risk factors for thyroid nodules and thyroid cancer; the results are expressed as ORs and 95% confidence intervals (CIs). Logistic regression analysis was performed to assess the risk factors for daily iodized salt intake in all physical examination subjects and thyroid nodule patients; the results are presented as ORs and 95% CIs. Multivariate linear regression analysis was performed to determine the risk factors for thyroid nodules, thyroid cancer, and daily iodized salt intake; for all enrolled subjects, the final risk assessment model was constructed using a stepwise method. SPSS 20.0 (IBM, Chicago, IL, USA) was used for statistical analyses. *P* < 0.05 was considered statistically significant.

## Results

### General data of the subjects

**Fig. 1:**
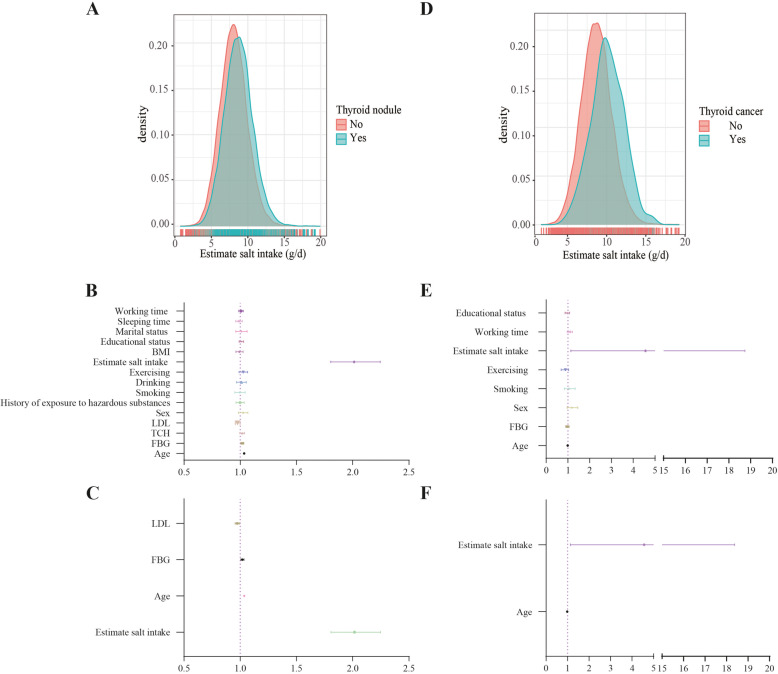
Analysis the risk factors of thyroid nodules and thyroid cancer. **A** Distribution of iodized salt intake for all medical examiners. **B** Risk factors for thyroid nodules. **C** Independent risk factors for thyroid nodules. **D** Distribution of iodized salt intake for patients with thyroid nodule **E** Risk factors for thyroid cancer. **F** Independent risk factors for thyroid cancer

Among the 51,637 included subjects, 20,784 were found to have thyroid nodules, accounting for 40.25% of the total (Supplemental Figure 1A). A total of 390 subjects were ultimately diagnosed with thyroid cancer, accounting for 0.76% of the total (Supplemental Figure 1B). Currently, the World Health Organization (WHO) recommends that daily salt intake should be less than 5 g [22], but only 3.59% of the subjects met this criterion (Supplemental Figure 1C). Compared with those subjects without thyroid nodules, age, fasting blood glucose (FBG), triglyceride (TCH), low-density lipoprotein (LDL), sex, history of exposure to hazardous substances, smoking and drinking history, estimate iodized salt intake, BMI, education level, marital status, sleep duration and working hours were the risk factors of thyroid nodules (Fig. 1A-B, Table 1). The independent risk factors were estimate iodized salt intake, age, FBG, LDL (Fig. 1C).

**Table 1:** Clinicopathologic characteristics among people with or without thyroid nodule

Variables	Non-nodule (*n* = 30,853)	Nodule (*n* = 20,784)	*p*
Age (y)	43.75 ± 11.51	48.68 ± 12.16	0.001
FBG (mmol/L)	5.53 ± 1.28	5.69 ± 1.50	< 0.001
TAG (mmol/L)	1.84 ± 1.78	1.84 ± 1.84	0.200
TCH (mmol/L)	5.01 ± 0.97	5.10 ± 1.01	< 0.001
HDL (mmol/L)	1.33 ± 0.30	1.34 ± 0.30	0.228
LDL (mmol/L)	2.84 ± 0.82	2.91 ± 0.85	< 0.001
Estimate iodized salt intake	8.08 ± 1.95	8.75 ± 1.98	< 0.001
Sex [n (%)]			< 0.001
Male	19,459 (63.1)	10,383 (50.0)	
Female	11,394 (36.9)	10,401 (50.0)	
Family history of cancer [n (%)]			0.175
No	29,975 (97.2)	20,234 (97.4)	
Yes	878 (2.8)	550 (2.6)	
History of exposure to hazardous substances [n (%)]			< 0.001
No	16,518 (53.5)	10,718 (51.6)	
Yes	14,335 (46.5)	10,066 (48.4)	
Smoking [n (%)]			0.024
No	23,337 (75.6)	15,539 (74.8)	
Yes	7516 (24.4)	5245 (25.2)	
Drinking [n (%)]			< 0.001
No	21,077 (68.3)	13,874 (66.8)	
Yes	9776 (31.7)	6910 (33.2)	
Exercising [n (%)]			< 0.001
No	11,306 (36.6)	8758 (42.1)	
Yes	19,547 (63.4)	12,026 (57.9)	
Estimate salt intake [n (%)]			< 0.001
≤ 5 g	1393 (4.5)	462 (2.2)	
> 5 g	29,460 (95.5)	20,322 (97.8)	
BMI [n (%)]			0.001
≤ 18.5	6106 (19.8)	4376 (21.1)	
18.5–24	21,730 (70.4)	14,524 (69.9)	
24–28	2538 (8.2)	1588 (7.6)	
> 28	479 (1.6)	296 (1.4)	
Educational status [n (%)]			< 0.001
Senior high school and below	10,110 (32.8)	9489 (45.9)	
Undergraduate college	15,479 (50.1)	9256 (44.5)	
Postgraduate and above	5264 (17.1)	2039 (9.9)	
Marital status [n (%)]			< 0.001
Single	2686 (8.6)	1203 (5.8)	
Married	27,331 (88.6)	18,905 (91.0)	
Divorced	603 (2.0)	400 (1.9)	
Windowed	233 (0.8)	276 (1.3)	
Sleeping time [n (%)]			0.006
< 5 h	2929 (9.5)	1954 (9.4)	
5-7 h	19,162 (62.1)	13,199 (63.5)	
7-9 h	8411 (27.3)	5422 (26.1)	
> 9 h	351 (1.1)	209 (1.0)	
Working time [n (%)]			0.007
< 4 h	3585 (11.6)	2625 (12.6)	
4-6 h	4931 (16.0)	3267 (15.8)	
6-8 h	14,024 (45.5)	9338 (44.9)	
> 8 h	8313 (26.9)	5554 (26.7)	

*Abbreviations*: *FBG* fasting blood glucose, *TAG* Triglyceride, *TCH* total cholesterol, *HDL* high density lipoprotein, *LDL* Low density lipoprotein

On the basis of the above results, we conducted a telephone follow-up with 20,784 patients with thyroid nodules and found that 390 of them were eventually diagnosed with thyroid cancer. Compared with subjects with noncancerous nodules, subjects diagnosed with thyroid cancer were of a lower age and had lower fasting blood glucose levels. Other relevant factors included daily iodized salt intake, sex, smoking, physical activity, BMI, education level, and working hours (Fig. 1D-E, Table 2). The independent risk factors were estimate iodized salt intake and age (Fig. 1F).

**Table 2:** Clinicopathologic characteristics among patients with or without thyroid cancer

Variables	Non-cancer (*n* = 20,394)	Cancer (*n* = 390)	*p*
Age (y)	48.72 ± 12.18	46.54 ± 11.11	0.036
FBG (mmol/L)	5.69 ± 1.50	5.58 ± 1.06	0.038
TAG (mmol/L)	1.84 ± 1.85	1.81 ± 1.53	0.621
TCH (mmol/L)	5.10 ± 1.01	4.97 ± 0.94	0.435
HDL (mmol/L)	1.34 ± 0.30	1.33 ± 0.29	0.528
LDL (mmol/L)	2.91 ± 0.85	2.81 ± 0.83	0.877
Estimate iodized salt intake	8.72 ± 1.96	10.11 ± 2.09	0.045
Sex [n (%)]			0.003
Male	10,217 (50.1)	166 (42.6)	
Female	10,177 (49.9)	224 (57.4)	
Family history of cancer [n (%)]			0.136
No	19,859 (97.4)	375 (96.2)	
Yes	535 (2.6)	15 (3.8)	
History of exposure to hazardous substances [n (%)]			0.750
No	10,520 (51. 6)	198 (50.8)	
Yes	9874 (48.4)	192 (49.2)	
Smoking [n (%)]			0.021
No	15,267 (74.9)	272 (69.7)	
Yes	5127 (25.1)	118 (30.3)	
Drinking [n (%)]			0.311
No	13,623 (66.8)	251 (64.4)	
Yes	6771 (33.2)	139 (35.6)	
Exercising [n (%)]			< 0.001
No	8510 (41.7)	248 (63.6)	
Yes	11,884 (58.3)	142 (36.4)	
Estimate salt intake [n (%)]			0.021
≤ 5 g	460 (2.3)	2 (0.5)	
> 5 g	19,934 (27.7)	388 (99.5)	
BMI [n (%)]			0.019
≤ 18.5	4269 (20.9)	107 (27.4)	
18.5–24	14,273 (70.0)	251 (64.4)	
24–28	1560 (7.7)	28 (7.2)	
> 28	292 (1.4)	4 (1.0)	
Educational status [n (%)]			< 0.001
Senior high school and below	9476 (45.5)	213 (54.6)	
Undergraduate college	9117 (44.7)	139 (35.6)	
Postgraduate and above	2001 (9.8)	38 (9.6)	
Marital status [n (%)]			0.318
Single	1183 (5.8)	20 (5.1)	
Married	18,542 (90.9)	363 (93.1)	
Divorced	397 (1.9)	3 (0.8)	
Windowed	272 (1.3)	4 (1.0)	
Sleeping time [n (%)]			0.347
< 5 h	1914 (9.4)	40 (10.3)	
5-7 h	12,967 (63.6)	232 (59.5)	
7-9 h	5307 (26.0)	115 (29.4)	
> 9 h	206 (1.0)	3 (0.8)	
Working time [n (%)]			0.009
< 4 h	2557 (12.6)	68 (17.4)	
4-6 h	3198 (15.7)	69 (17.7)	
6-8 h	9186 (45.0)	152 (39.0)	
> 8 h	5453 (26.7)	101 (25.9)	

*Abbreviations*: *FBG* fasting blood glucose, *TAG* Triglyceride, *TCH* total cholesterol, *HDL* high density lipoprotein, *LDL* Low density lipoprotein

### Correlation between daily iodized salt intake and thyroid nodules and thyroid cancer

To further investigate the correlation between daily iodized salt intake and thyroid nodules and thyroid cancer, variables were introduced through stepwise logistic regression analysis. Compared with that in subjects without thyroid nodules, when only daily iodized salt intake was included as a variable for analysis of subjects with thyroid nodules, the OR was 2.072 [95% CI: 1.863–2.305, *p* < 0.001 (Model 1, Table 3)]. With the introduction of other variables, daily iodized salt intake was always a risk factor that increased the risk of thyroid nodules (Model 2–8, Table 3).

**Table 3:** Odds ratios and 95% confidence intervals to thyroid nodule

	Non-nodule	Nodule	*p*
Model 1	1.00	2.072 (1.863–2.305)	< 0.001
Model 2	1.00	2.074 (1.854–2.307)	< 0.001
Model 3	1.00	2.072 (1.857–2.311)	< 0.001
Model 4	1.00	2.093 (1.876–2.336)	< 0.001
Model 5	1.00	2.089 (1.873–2.311)	< 0.001
Model 6	1.00	2.075 (1.858–2.318)	< 0.001
model 7	1.00	2.064 (1.848–2.306)	< 0.001
model 8	1.00	2.064 (1.848–2.306)	< 0.001

Model 1 was for estimate iodized salt intake

Model 2 was adjusted for BMI

Model 3 was further adjusted for age and sex

Model 4 was further adjusted for FBG

Model 5 was further adjusted for TCH and LDL

Model 6 was further adjusted for history of exposure to hazardous substances, smoking, drinking and exercising

Model 7 was further adjusted for educational status and marital status

Model 8 was further adjusted for sleeping time and working time

In the subjects with thyroid cancer, compared with those with thyroid nodules but without cancer, when only daily iodized salt intake was included as a variable for analysis, the OR was 4.477 [95% CI. 1.112–18.019, *p* = 0.035 (Model 1, Table 4)]. After introducing other variables stepwise, daily iodized salt intake was always a risk factor for thyroid cancer (Model 2–7, Table 4).

**Table 4:** Odds ratios and 95% confidence intervals to thyroid cancer

	Non-cancer	Cancer	*p*
Model 1	1.00	4.477(1.112–18.019)	0.035
Model 2	1.00	4.454(1.106–17.928)	0.036
Model 3	1.00	4.615(1.146–18.583)	0.031
Model 4	1.00	4.599(1.142–18.520)	0.032
Model 5	1.00	5.722(1.419–23.071)	0.014
Model 6	1.00	4.673(1.159–18.840)	0.030
model 7	1.00	4.684(1.161–18.888)	0.030

Model 1 was for estimate iodized salt intake

Model 2 was adjusted for BMI

Model 3 was further adjusted for age and sex

Model 4 was further adjusted for FBG

Model 5 was further adjusted for smoking and exercising

Model 6 was further adjusted for educational status

Model 7 was further adjusted for working time

**Fig. 2:**
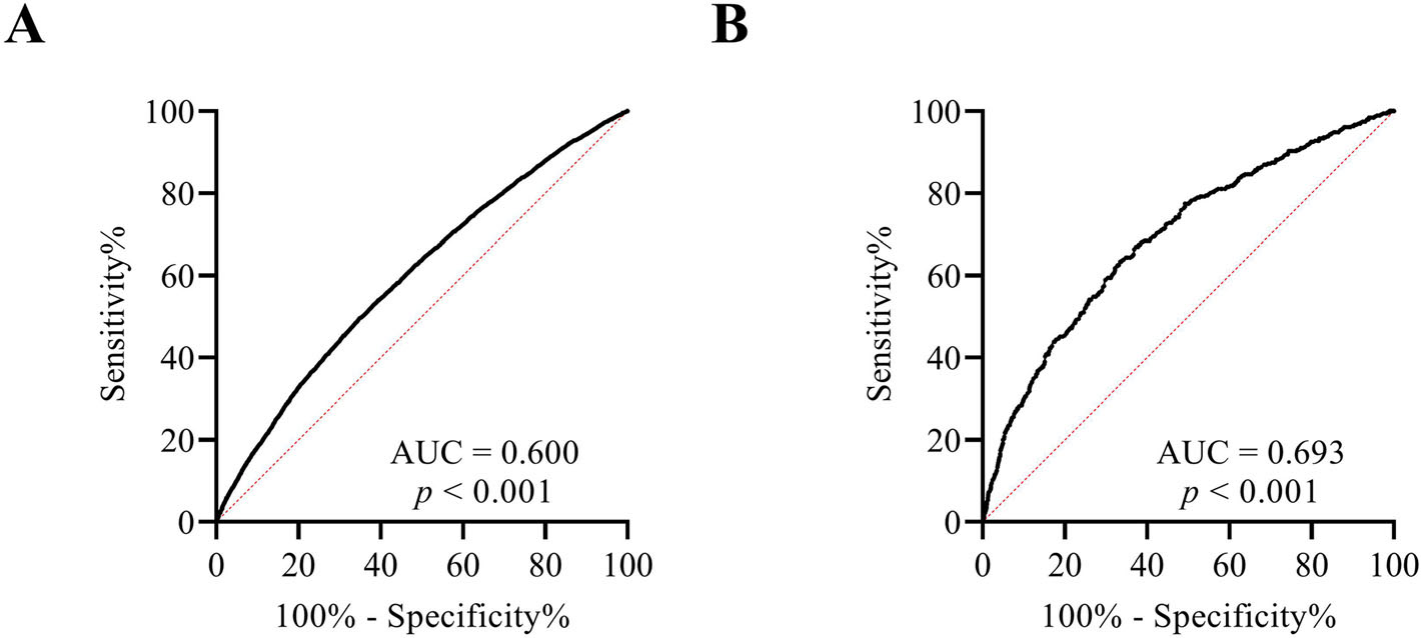
Value of daily iodized salt intake in the diagnosis of thyroid nodules and thyroid cancer. **A** Thyroid nodules. **B** Thyroid cancer

Because daily iodized salt intake is not only an independent risk factor for thyroid nodules but also an independent risk factor for thyroid cancer, we further evaluated the feasibility of daily iodized salt intake as a novel indicator for the clinical diagnosis of thyroid nodules and thyroid cancer. Notably, daily iodized salt intake can be used as an important reference indicator for the diagnosis of both thyroid nodules and thyroid cancer, with areas under the ROC curve (AUCs) of 0.600 and 0.693, respectively ((Fig. 2A-B).

### The combined effects of daily iodized salt intake and age, physical activity, and education level on the risk of thyroid nodules and thyroid cancer

In previous studies, we found that daily iodized salt intake, age, physical activity, and education level were all independent risk factors for thyroid nodules and thyroid cancer. Therefore, we further attempted to use these indicators in a combined analysis (Tables 5, 6). Compared with subjects younger than 60 years old with a daily intake of iodized salt not exceeding 5 g, regardless of an increase in age or daily iodized salt increase, subjects older than 60 years old with a daily intake of iodized salt not exceeding 5 g had a higher risk of thyroid nodules; the risk of thyroid nodules was highest among those who were older than 60 years old and whose daily iodized salt intake exceeded 5 g (OR: 4.88, 95% CI: 4.29–5.54, *p* < 0.001). In the combined analysis with thyroid cancer, an increased risk of cancer was observed only when daily iodized salt intake exceeded 5 g (OR: 3.84, 95% CI: 1.95–15.46, *p* = 0.042). Compared with those who did not exercise and had a daily iodized salt intake of no more than 5 g, those who did regularly participate in physical activity exhibited a reduced risk of thyroid nodules. Therefore, although the daily intake of more than 5 g of iodized salt increased the risk of thyroid nodules, the risk of thyroid nodules associated with a daily intake of iodized salt greater than 5 g can be partially offset by physical activity in this population (OR: 1.12, 95% CI: 0.97–1.28, *p* = 0.111); similar results were also observed in the combined thyroid cancer analysis. Compared with those who had an education level of high school or below and daily iodized salt intake no more than 5 g, those with higher education levels exhibited a reduced risk of thyroid nodules; however, a daily intake of iodized salt greater than 5 g increased the risk of thyroid nodules. Therefore, compared with that in subjects with a high school education or lower, the risk of thyroid nodules was lower among those subjects with a postgraduate education or higher and with a daily iodized salt intake greater than 5 g (OR: 0.79, 95% CI: 0.66–0.93, *p* = 0.005), indicating that the increased risk of thyroid nodules caused by daily iodized salt intake greater than 5 g can be offset by education level. In the combined analysis with thyroid cancer, daily iodized salt intake and education level were not statistically significant.

**Table 5:** Joint association between estimate iodized salt intake and age/ exercising/ educational status on risk of thyroid nodule

	Non-nodule	Nodule	OR (95% CI)	*p*
	n (%)	n (%)		
Estimate iodized salt intake and Age				
Estimate iodized salt intake ≤5 g				
Age ≤ 60y	1270 (4.12)	368 (1.77)	1.00	
Age > 60y	123 (0.40)	94 (0.45)	2.64 (1.97–3.53)	< 0.001
Estimate iodized salt intake > 5 g				
Age ≤ 60y	27,144 (87.98)	17,050 (82.03)	2.16 (1.92–2.43)	< 0.001
Age > 60y	2316 (7.51)	3272 (15.74)	4.88 (4.29–5.54)	< 0.001
Estimate iodized salt intake and exercising				
Estimate iodized salt intake ≤5 g				
No	576 (1.87)	327 (1.57)	1.00	
Yes	817 (2.65)	135 (0.65)	0.29 (0.23–0.37)	< 0.001
Estimate iodized salt intake > 5 g				
No	10,730 (34.78)	8431 (40.56)	1.38 (1.21–1.59)	< 0.001
Yes	18,730 (60.71)	11,891 (57.21	1.12 (0.97–1.28)	0.111
Estimate iodized salt intake and educational status				
Estimate iodized salt intake ≤5 g				
Senior high school and below	429 (1.39)	217 (1.04)	1.00	
Undergraduate college	724 (2.35)	200 (0.96)	0.55 (0.44–0.69)	< 0.001
Postgraduate and above	240 (0.78)	45 (0.22)	0.37 (0.26–0.53)	< 0.001
Estimate iodized salt intake > 5 g				
Senior high school and below	9681 (31.38)	9272 (44.61)	1.89 (1.60–2.24)	< 0.001
Undergraduate college	14,755 (47.82)	9056 (43.57)	1.23 (1.03–1.43)	0.022
Postgraduate and above	5024 (16.28)	1994 (9.59)	0.79 (0.66–0.93)	0.005

**Table 6:** Joint association between estimate iodized salt intake and age/ exercising/ educational status on risk of thyroid cancer

	Non-cancer	Cancer	OR (95% CI)	P
	n (%)	n (%)		
Estimate iodized salt intake and Age				
Estimate iodized salt intake ≤5 g				
Age ≤ 60y	366 (1.79)	2 (0.51)	1.00	
Age > 60y	94 (0.46)	0 (0.00)	1.00 (0.99–1.00)	0.474
Estimate iodized salt intake > 5 g				
Age ≤ 60y	16,700 (81.89)	350 (89.74)	3.84 (1.95–15.46)	0.042
Age > 60y	3234 (15.86)	38 (9.75)	2.15 (0.52–8.95)	0.281
Estimate iodized salt intake and exercising				
Estimate iodized salt intake ≤5 g				
No	325 (1.59)	2 (0.51)	1.00	
Yes	135 (0.66)	0 (0.00)	0.99 (0.99–1.00)	0.362
Estimate iodized salt intake > 5 g				
No	8185 (40.13)	246 (63.08)	4.88 (1.21–19.73)	0.014
Yes	11,749 (57.61)	142 (36.41)	1.96 (0.48–7.96)	0.336
Estimate iodized salt intake and educational status				
Estimate iodized salt intake ≤5 g				
Senior high school and below	215 (1.05)	2 (0.51)	1.00	
Undergraduate college	200 (0.98)	0 (0.00)	0.99 (0.98–1.00)	0.174
Postgraduate and above	45 (0.22)	0 (0.00)	0.99 (0.98–1.00)	0.518
Estimate iodized salt intake > 5 g				
Senior high school and below	9061 (44.43)	211 (54.10)	2.50 (0.62–10.14)	0.183
Undergraduate college	8917 (43.72)	139 (35.64)	1.68 (0.41–6.81)	0.466
Postgraduate and above	1956 (9.59)	38 (9.74)	2.09 (0.50–8.72)	0.302

### Factors associated with estimated iodized salt intake

**Fig. 3:**
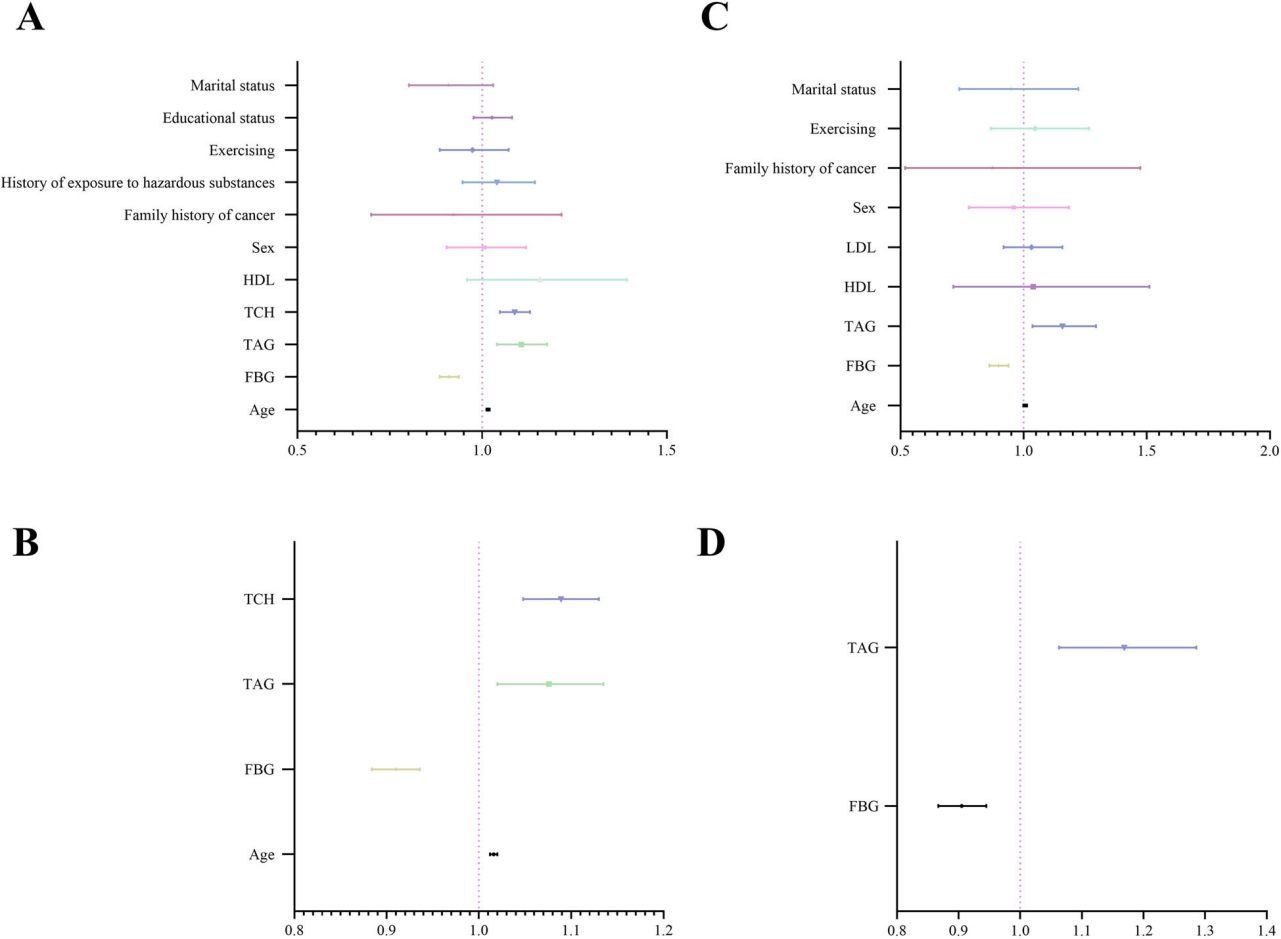
Multivariate linear regression analysis of daily iodized salt intake. **A** Risk factors affecting salt intake in all study populations. **B** Independent risk factors affecting salt intake in all study populations. **C** Risk factors affecting salt intake in the population with thyroid nodes. **D** Independent risk factors affecting salt intake in the population with thyroid nodules

The above studies showed that a daily iodized salt intake greater than 5 g was an independent risk factor for both thyroid nodules and thyroid cancer. Therefore, we further investigated the risk factors that could influence daily iodized salt intake. First, we analyzed which factors were related to a daily iodized salt intake greater than 5 g. The results showed that age, triglycerides, total cholesterol, history of exposure to hazardous substance, physical activity, and marital status were risk factors for a daily iodized salt intake greater than 5 g. Fasting blood glucose, sex, family history of tumors, and education level were protective factors against a daily iodized salt intake greater than 5 g (Table 7, Fig. 3A, Suppl Fig 2). Further linear regression analysis showed that independent risk factors for daily iodized salt intake greater than 5 g included age, fasting blood glucose, triglycerides, total cholesterol, sex, family history of tumors, history of exposure to hazardous substance, physical activity, education level, and marital status (Fig. 3B). On this basis, we also conducted a subgroup analysis of subjects with thyroid nodules. We found that age, triglycerides, physical activity, and marital status were risk factors for a daily iodized salt intake greater than 5 g, whereas fasting blood glucose, high-density lipoprotein (HDL), LDL, sex, and family history of tumors were protective factors against a daily iodized salt intake greater 5 g (Table 8, Fig. 3C, Suppl Fig 3). Linear regression analysis indicated that the independent risk factors for a daily iodized salt intake greater than 5 g in patients with thyroid nodules were age, triglycerides, family history of tumors, physical activity, and marital status (Fig. 3D). These results indicated that independent of the specific population with thyroid nodules, age, triglycerides, and family history of tumors, physical activity, and marital status were independent risk factors for a daily iodized salt intake greater than 5 g. Improvements in these areas of daily life can help reduce daily iodized salt intake.

**Table 7:** Clinicopathologic characteristics among all people grouped by estimate iodized salt intake

	Salt intake (≤ 5 g, *n* = 1855)	Salt intake [> 5 g, *n* = 49,782]	*p*
Age (y)	43.70 ± 13.44	45.82 ± 11.96	< 0.001
FBG (mmol/L)	5.69 ± 1.96	5.59 ± 1.35	< 0.001
TAG (mmol/L)	1.64 ± 1.55	1.84 ± 1.82	< 0.001
TCH (mmol/L)	4.92 ± 1.05	5.05 ± 0.98	0.003
HDL (mmol/L)	1.36 ± 0.32	1.34 ± 0.30	< 0.001
LDL (mmol/L)	2.79 ± 0.85	2.87 ± 0.83	0.283
Sex [n (%)]			< 0.001
Male	968 (52.2)	28,874 (58.0)	
Female	887 (47.8)	20,908 (42.0)	
Family history of cancer [n (%)]			< 0.001
No	1750 (94.3)	48,459 (97.3)	
Yes	105 (5.7)	1323 (2.7)	
History of exposure to hazardous substances [n (%)]			< 0.001
No	1623 (87.5)	25,613 (51.5)	
Yes	232 (12.5)	24,169 (48.5)	
Smoking [n (%)]			0.339
No	1414 (76.2)	37,462 (75.3)	
Yes	441 (23.8)	12,320 (24.7)	
Drinking [n (%)]			0.326
No	1275 (68.7)	33,676 (67.6)	
Yes	580 (31.3)	16,106 (32.4)	
Exercising [n (%)]			< 0.001
No	903 (48.7)	19,161 (38.5)	
Yes	952 (51.3)	30,621 (61.5)	
BMI [n (%)]			0.815
≤ 18.5	379 (20.4)	10,103 (20.3)	
18.5–24	1302 (70.2)	34,952 (70.2)	
24–28	151 (8.1)	3975 (8.0)	
> 28	23 (1.2)	752 (1.5)	
Educational status [n (%)]			0.015
Senior high school and below	646 (34.8)	18,953(38.0)	
Undergraduate college	924 (49.8)	23,811 (47.8)	
Postgraduate and above	285 (15.4)	7018 (14.1)	
Marital status [n (%)]			< 0.001
Single	254 (13.7)	3635 (7.3)	
Married	1544 (83.2)	44,692 (89.8)	
Divorced	42 (2.3)	961 (1.9)	
Windowed	15 (0.8)	494 (1.0)	
Sleeping time [n (%)]			0.894
< 5 h	167 (9.0)	4716 (9.5)	
5-7 h	1174 (63.3)	31,187 (62.6)	
7-9 h	493 (26.6)	13,340 (26.8)	
> 9 h	21 (1.1)	539 (1.1)	
Working time [n (%)]			0.602
< 4 h	241 (13.0)	5969 (12.0)	
4-6 h	294 (15.8)	7904 (15.9)	
6-8 h	822 (44.3)	22,540 (45.3)	
> 8 h	498 (16.8)	13,369 (26.9)	

*Abbreviations*: *FBG* fasting blood glucose, *TAG* Triglyceride, *TCH* total cholesterol, *HDL* high density lipoprotein, *LDL* Low density lipoprotein

**Table 8:** Clinicopathologic characteristics among patients with thyroid nodule grouped by estimate iodized salt intake

	Salt intake (≤ 5 g, *n* = 462)	Salt intake (> 5 g, *n* = 20,322)	*p*
Age (y)	47.91 ± 14.52	48.69 ± 12.10	< 0.001
FBG (mmol/L)	5.98 ± 2.43	5.69 ± 1.47	< 0.001
TAG (mmol/L)	4.97 ± 1.10	5.10 ± 1.00	0.041
TCH (mmol/L)	1.63 ± 1.53	1.84 ± 1.85	0.057
HDL (mmol/L)	1.36 ± 0.34	1.34 ± 0.30	< 0.001
LDL (mmol/L)	2.83 ± 0.93	2.91 ± 0.84	0.032
Sex [n (%)]			0.032
Male	208 (45.0)	10,175 (50.1)	
Female	254 (55.0)	10,147 (49.9)	
Family history of cancer [n (%)]			< 0.001
No	400 (86.6)	19,834 (97.6)	
Yes	62 (13.4)	488 (2.4)	
History of exposure to hazardous substances [n (%)]			0.438
No	230 (49.8)	10,488 (51.6)	
Yes	232 (50.2)	9834 (48.4)	
Smoking [n (%)]			0.173
No	358 (77.5)	15,181 (74.7)	
Yes	104 (22.5)	5141 (25.3)	
Drinking [n (%)]			0.889
No	307 (66.5)	13,567 (66.8)	
Yes	155 (33.5)	6755 (33.2)	
Exercising [n (%)]			< 0.001
No	327 (70.8)	8431 (41.5)	
Yes	135 (29.2)	11,891 (58.5)	
BMI [n (%)]			0.064
≤ 18.5	102 (22.0)	4274 (21.0)	
18.5–24	303 (65.6)	14,221 (70.0)	
24–28	47 (10.2)	1541 (7.6)	
> 28	10 (2.2)	286 (1.4)	
Educational status [n (%)]			0.841
Senior high school and below	217 (47.0)	9272 (45.6)	
Undergraduate college	200 (43.3)	9056 (44.6)	
Postgraduate and above	45 (9.7)	1994 (9.8)	
Marital status [n (%)]			< 0.001
Single	50 (10.8)	1153 (5.7)	
Married	395 (85.5)	18,510 (91.1)	
Divorced	9 (1.9)	391 (1.9)	
Windowed	8 (1.7)	268 (1.3)	
Sleeping time [n (%)]			0.744
< 5 h	39 (8.4)	1915 (9.4)	
5-7 h	295 (63.9)	12,904 (63.5)	
7-9 h	125 (27.1)	5297 (26.1)	
> 9 h	3 (0.6)	206 (1.0)	
Working time [n (%)]			0.367
< 4 h	55 (11.9)	2570 (12.6)	
4-6 h	84 (18.2)	3183 (15.7)	
6-8 h	211 (45.7)	9127 (44.9)	
> 8 h	112 (24.2)	5442 (26.8)	

*Abbreviations*: *FBG* fasting blood glucose, *TAG* Triglyceride, *TCH* total cholesterol, *HDL* high density lipoprotein, *LDL* Low density lipoprotein

## Discussion

Through a retrospective analysis of data of 51,637 individuals who underwent a physical examination in the Hunan region, this study found that the daily iodized salt intake of most subjects in this region was significantly higher than the recommended intake by the WHO and that a daily iodized salt intake greater than 5 g is an independent risk factor for the occurrence of thyroid nodules and even thyroid cancer. Factors such as age, triglycerides, family history of tumors, physical activity, and marital status were all independent risk factors affecting daily iodized salt intake, both in general subjects who underwent a physical examination and in subjects with thyroid nodules,

Hunan is located inland in China, and the intake of seafood is relatively low; the intake of iodine is almost entirely from iodized salt. In other studies, daily iodized salt intake was often determined through the subjective feelings of taste (light, normal, and salty) [23, 24], and the data did not reflect the actual situation. In this study, daily salt intake was estimated by the spot urine sample, result in more accurate data. And previous studies have shown that the values obtained by spot urine methods correlated highly with daily salt intake, can be used to estimate daily iodized salt intake [25, 26].

Excessive daily salt intake is closely related to diseases such as hypertension, calcium loss, and kidney diseases [27, 28]. Therefore, the daily salt intake recommended by the WHO is less than 5 g. Whether the intake of iodized salt increases the risk of thyroid nodules and the risk of thyroid cancer remains controversial in academia [29, 30]. In this study, a retrospective analysis of big data indicated that when daily iodized salt intake exceeds 5 g, there was an increased risk of thyroid nodules and thyroid cancer. This result supports the scientific validity of the WHO recommendations.

In this study, patients with thyroid nodules had higher blood glucose and blood lipid levels, indicating that patients with thyroid nodules were more prone to metabolic disorders. In the population with thyroid nodules, the average age of the patients with noncancerous nodules was 48.72 years, and the average age of patients with cancerous nodules was 46.54, indicating a trend toward a younger age, which is consistent with the trend for thyroid disease, i.e., presentation a younger ages. In addition, we found that with the increase in education level, salt intake decreased, and the incidence of thyroid nodules also decreased. Thus, a higher education level is conducive to acquiring relevant health knowledge, paying more attention to one’s own health, and moderating salt intake, which demonstrates, from a different perspective, that controlling salt intake is very beneficial for the prevention of thyroid nodules, in addition to reducing the occurrence of hypertension and kidney disease. In the combined analysis of daily iodized salt intake and education level, with the same daily intake of iodized salt, with an increase in education level, the risk of thyroid nodules was reduced, suggesting that education can reduce the risk of thyroid nodules through other factors, in addition to influencing daily iodized salt intake. Marriage status showed a different trend: in all populations who underwent a physical examination and in those with thyroid nodules, married people always tended to have a greater intake of iodized salt, but the incidence of thyroid nodules decreased. We speculate that this is caused by other confounding factors. Finally, we observed that individuals with a family history of tumors tended to have lower daily iodized salt intake, a result that might be related to the fact that families of cancer patients are more likely to choose healthy lifestyles, including decreasing daily iodized salt intake. In the combined analysis of daily iodized salt intake and other factors, we found that physical activity could partially eliminate the increased risk of thyroid nodules and thyroid cancer associated with excessive daily iodized salt intake; therefore, we strongly recommend moderate exercise.

Although this study had a large sample size, there are still limitations. This study is a single-center retrospective cross-sectional analysis, and the observed number of thyroid cancer cases was small, only 390, which may affect the reliability of the results to some extent. In addition, the limited enrollment may affect the general applicability of the results to a certain extent. Nevertheless, we can still conclude that currently, the vast majority of the population in inland hilly areas of China have a daily iodized salt intake that is excessive, and the excessive intake of iodized salt will increase the risk of thyroid nodules and thyroid cancer. Limiting salt is imperative; improvements in lifestyles such as salt-restricted diets, moderate exercise, and health knowledge can reduce the risk of thyroid nodules and thyroid cancer.

## Supplementary Information

**Additional file 1: Suppl Fig 1.** General data of the subjects. **A**. The proportion of thyroid nodules. **B**. The proportion of thyroid cancer. **C**. The proportion of daily iodized salt intake.

**Additional file 2: Suppl Fig 2.** Daily iodized salt intake in medical examiners.

**Additional file 3: Suppl Fig 3.** Daily iodized salt intake in patients with thyroid nodule.

## Data Availability

The data and materials of this study are available from the corresponding author for reasonable requests.

## Abbreviations

OR: Odds ratio
CI: Confidence interval
BMI: Body mass index
LDL: Low-density lipoprotein
HDL: High-density lipoprotein
