# Supplementary figures and images for: Analysis of the correlation between high iodized salt intake and the risk of thyroid nodules: a large retrospective study
Source: BMC Cancer. 2021 Sep 7;21:1000. doi: 10.1186/s12885-021-08700-z (PMC8425165; doi:10.1186/s12885-021-08700-z)

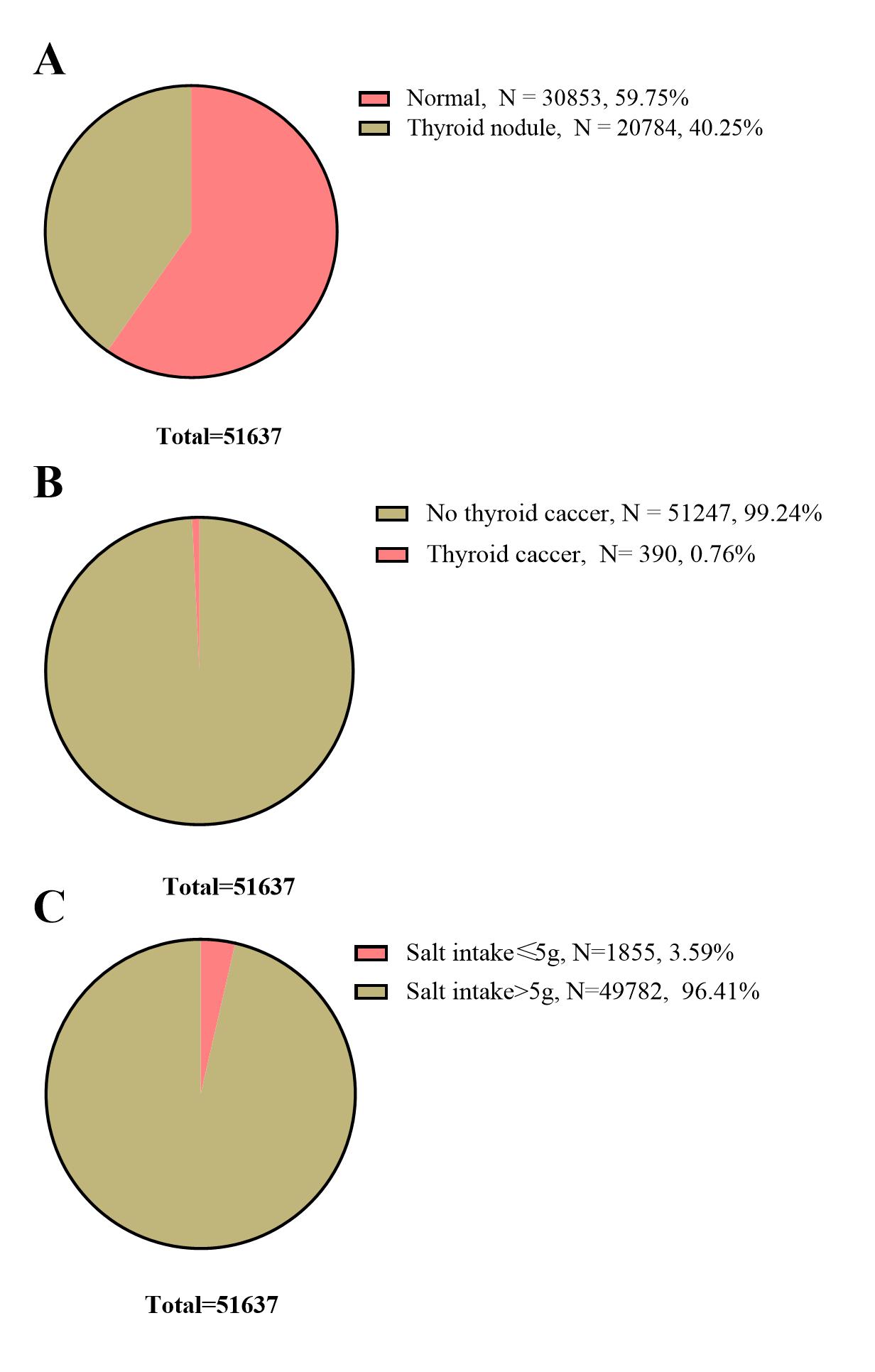

Supplement: Supplementary file 1 — Additional file 1: Suppl Fig 1. General data of the subjects. A. The proportion of thyroid nodules. B. The proportion of thyroid cancer. C. The proportion of daily iodized salt intake. [file 12885_2021_8700_MOESM1_ESM.jpg]

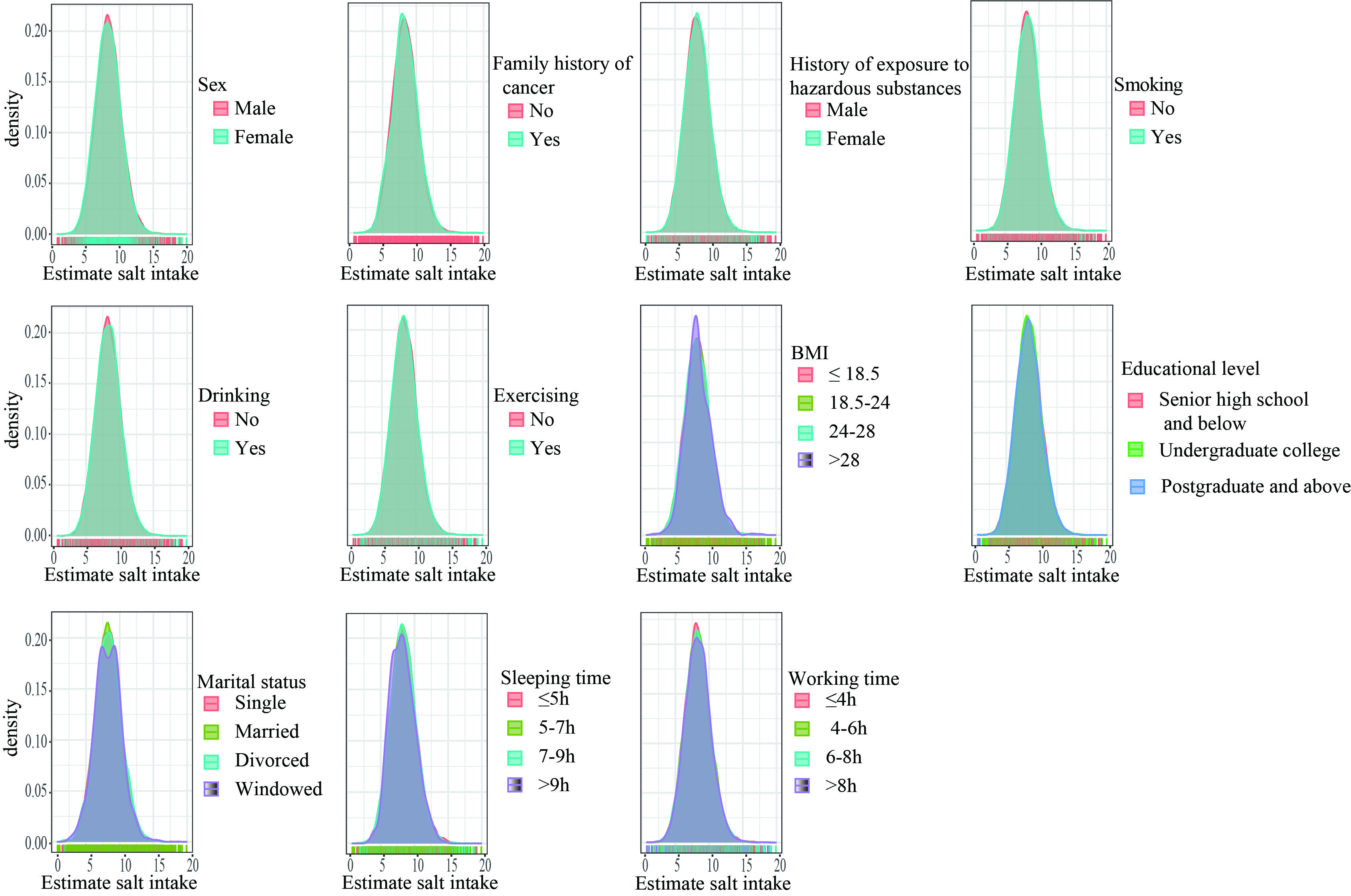

Supplement: Supplementary file 2 — Additional file 2: Suppl Fig 2. Daily iodized salt intake in medical examiners. [file 12885_2021_8700_MOESM2_ESM.jpg]

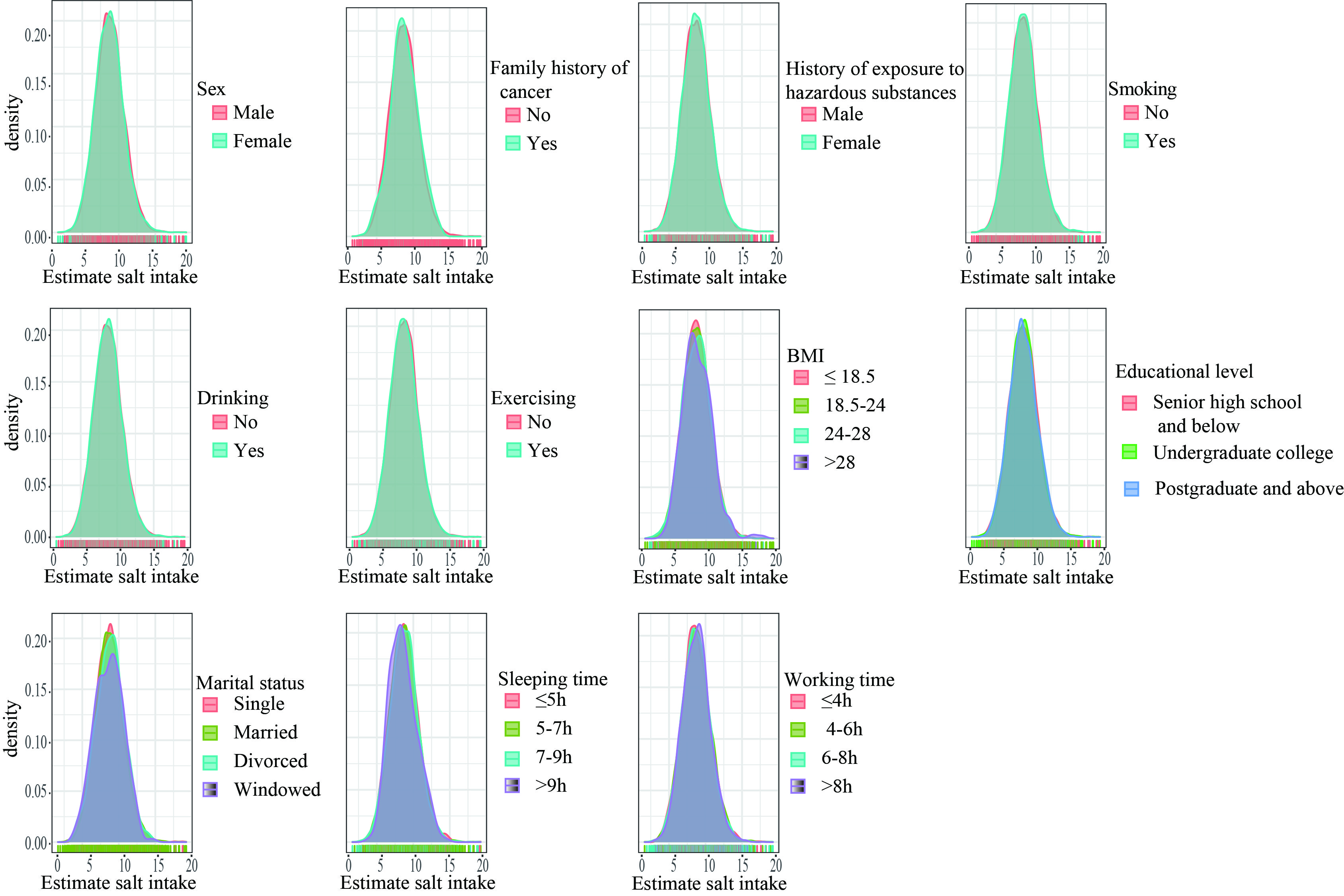

Supplement: Supplementary file 3 — Additional file 3: Suppl Fig 3. Daily iodized salt intake in patients with thyroid nodule. [file 12885_2021_8700_MOESM3_ESM.jpg]
